# An exploratory randomised controlled trial of a web-based integrated bipolar parenting intervention (IBPI) for bipolar parents of young children (aged 3–10)

**DOI:** 10.1186/s12888-015-0505-y

**Published:** 2015-06-06

**Authors:** Steven Jones, Laura D Wainwright, Jelena Jovanoska, Helen Vincent, Peter J Diggle, Rachel Calam, Rob Parker, Rita Long, Debbie Mayes, Matthew Sanders, Fiona Lobban

**Affiliations:** Spectrum Centre for Mental Health Research, Faculty of Health and Medicine, Lancaster University, LA1 4YT Lancaster, UK; CHICAS, Faculty of Health and Medicine Lancaster University, Lancaster, LA1 4YT UK; School of Psychological Sciences, University of Manchester, Manchester, M13 9PL UK; Parenting and Family Support Centre, School of Psychology, University of Queensland, Brisbane, QLD 4072 Australia

## Abstract

**Background:**

Communication, impulse control and motivation can all be affected by Bipolar Disorder (BD) making consistent parenting more difficult than for parents without mental health problems. Children of parents with BD (CPB) are at significantly increased risk of a range of mental health issues including Attention Deficit Hyperactivity Disorder (ADHD), anxiety, depression, substance use, and sleep disorders. Furthermore, CPB are also at elevated risk for BD compared to the general population. This paper describes the rationale and protocol for a pilot randomised controlled trial (RCT) designed to assess the feasibility and acceptability of a new online intervention providing interactive psychoeducational information and parenting support for parents with BD.

**Methods and design:**

This article describes a single-blind randomised controlled trial comparing an Integrated Bipolar Parenting Intervention (IBPI) in addition to treatment as usual (TAU) with TAU alone. Participants will be recruited from across the UK from mental health services and through self-referral. The primary outcome of the study is the feasibility and acceptability of IBPI as indicated by recruitment to target, use of the intervention site, and retention to follow-up. Parents with BD allocated to the IBPI condition will have access to the intervention for 16 weeks. Effect size estimates will be obtained with respect to child behaviour, parenting skills and measures of parental mental health using measures taken at baseline (0), and at 16, 24, 36, and 48 weeks post randomization.

**Discussion:**

This is the first randomised controlled trial of an integrated bipolar disorder parenting intervention. The benefits and challenges of delivering this online intervention, and evaluation using online RCT methodology are discussed.

**Trial registration:**

Current Controlled Trials ISRCTN75279027 Registered 12 August 2013

## Background

Bipolar Disorder (BD), which has a prevalence rate of approximately 1 to 1.5 % [1] is characterised by recurrent periods of high mood (mania) and low mood (depression). Difficulties with work-related performance, financial issues, social/leisure activities and social/family relationships are common [2], though positive experiences of BD have also been identified [3]. Overall, BD tends to have a significant negative impact on the individual, family, and society, with an estimated annual financial cost in England alone of £5.2 billion [4].

Many individuals with mental health problems are also parents. An Australian study estimated that 23 % of children live in families with at least one parent with a mental health illness and 20 % of users of mental health services have dependent children [5]. The Royal College of Psychiatrist’s report ‘Parents as patients: supporting the needs of patients who are parents and their children’ [6] has highlighted the potential impact of parental mental health issues on children, especially severe mental illness, such as bipolar disorder. Parenting can be a particular challenge for parents who are living with bipolar disorder, as there can be difficulties with sleep/wake cycles, impulse control, motivation and communication [7,8] which can impact on their ability to consistently engage in adaptive parenting.

There is now consistent evidence that children of parents with BD (CPB) are vulnerable to a wide range of behavioural and emotional conditions including Attention Deficit Hyperactivity Disorder (ADHD), anxiety, depression, substance use, and sleep disorders, in addition to higher rates of BD compared to the general population [9-11]. However, it is possible that parenting programmes, designed to help parents to encourage desirable behaviour and cope with challenging behaviour in their children may be effective in enhancing the confidence and skills of bipolar parents, and in doing so, can help ameliorate these vulnerabilities. Parenting programmes are delivered in a range of formats from group settings to online and typically build on parenting strengths rather than highlighting weaknesses. There is evidence that such programmes can effectively reduce child problem behaviours including conduct, antisocial behaviour and attention deficit disorders [12-14].

A particular parenting programme (Triple P), developed by one of the authors (MS) has been shown to be effective in reducing problem behaviours and in improving both parenting skills and parental wellbeing [15]. In addition, there is evidence that in depressed mothers Triple P led to reductions in both maternal depression and in problem behaviour in their children [16]. Although there are Triple P studies which have included teenagers, the majority of trials have focussed on parents of pre-teenage children typically aged 2–3 to 9–11 [15].

There is a high demand for both structured psychological interventions for mental health issues [17] as recommended by National Institute for Clinical Excellence [18] and for community based parenting support [19,20]. However many individuals struggle to access either of these types of intervention due to lack of suitably trained therapists. The internet (currently used by 73 % of the UK population [21]) provides an alternative to face to face interventions offering the potential to deliver self-management help flexibly (at the time and location chosen by the client), cheaply and without the stigma that some people feel is associated with seeking support from mental health services [22].

A pilot study by our team has shown that providing simple psychoeducational information on BD along with an early online version of the Triple P Positive Parenting Program led to significant improvements in child behaviour and parenting [23]. The present study builds on this work using a new interactive self-management site for BD developed for the study and integrated with the most recent version of Triple P Online, [24] to provide a web-based integrated bipolar parenting intervention (IBPI).

This paper describes the rationale and protocol for a pilot randomised controlled trial (RCT) of IBPI designed to assess how feasible it is to implement an online parenting intervention for parents with BD. The study will therefore evaluate as primary outcomes, recruitment into the study and consent to participate, retention to the intervention and to follow-up assessments (both arms). The trial will also provide preliminary estimates of the impact of the intervention on child behaviour, parenting skill and confidence, parental mood (including time to further mood episodes) and family functioning.

## Method

This trial is conducted by a multidisciplinary team of researchers, clinicians, service users, and a statistician across academic institutions and NHS Trusts in the United Kingdom. This study was reviewed and approved by the UK NHS Ethics Committee process (REC ref: 12/NW/0749).

### Objective

To determine the feasibility and acceptability of an online integrated parenting intervention for bipolar disorder compared with treatment as usual.

Main research questions:

- To demonstrate feasibility of recruitment and consenting procedures, adherence to intervention and retention to both arms of the trial across assessment, intervention and 16, 24, 36 and 48 week follow up periods.
- To provide parameter estimates of clinical outcomes with respect to, child behaviour, parenting skills, mood, and family functioning.

### Trial design

A rater-blind randomised controlled trial which compares sixteen weeks of access to an online integrated bipolar parenting intervention (IBPI) plus treatment as usual (TAU) with TAU alone. The trial is based in the United Kingdom. Randomisation will be carried out by the independent Clinical Trials Unit at The Christie NHS Foundation Trust, Manchester.

### Sample size

The chief purpose of this trial is to evaluate the feasibility and acceptability of providing IBPI, thus a formal power calculation for a test comparing treatment groups is not appropriate.

The primary outcome, to demonstrate feasibility, will be recruitment into the trial and the proportion of completers of the intervention and of assessments at 16, 24, 36 and 48 weeks follow-up. Thirty participants per group, i.e. 60 in total, is sufficient to reliably determine the primary feasibility outcomes for the trial. We therefore aim to recruit N = 100 across both arms of the study to allow for a potential drop-out rate of up to 35-40 % based on an average of attrition rates across 4 relevant studies of web-based interventions for affective and/or parenting issues [20,24-26].

### Recruitment

Participants will be recruited in two ways:

1. Clinician Referral Route

Seventeen National Health Service (NHS) Trusts across the UK are taking part in this study. Community mental health teams, out-patient clinics, GP surgeries, primary care mental health teams and voluntary services will identify potential participants. Care coordinators, research nurses and research development officers will be approached in order to contact potential participants in the first instance. When recruiting in community mental health teams and voluntary services, a member of the research team will present an outline of the study and will provide written material about the study. Potential participants will be offered a participant information sheet by their care co-ordinators or the research team, outlining the study and their role should they wish to take part.

2. Self-Referral Routes

The study will be advertised in the media using social networks, such as Facebook, Twitter, relevant online forums and via adverts in local press. Posters and leaflets will be distributed in both NHS and non NHS sites such as relevant third sector bases (i.e. mental health organisations and parenting advice or children’s centres) to maximise participant access. There will be a significant focus on online recruitment. Due to the nature of the study, it is assumed that potential participants seeking advice online are more likely to engage with such an intervention.

**Figure 1:**
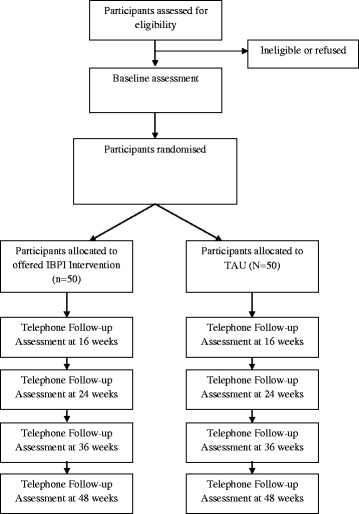
CONSORT diagram showing design of the study

Clinicians will be informed of a participant’s involvement in the study subject to participant consent. If a participant does not wish for their primary or secondary care clinician to be informed about their involvement in the study, this does not prevent their participation as long as they consent to their clinician being contacted should they be a significant risk to themselves or others during the study. Figure 1 gives an outline of the participant flow through the study.

Participants will be asked to express an interest in the study which will act as a ‘run in period’ before consent, so that any potential drop outs occur prior to consent to minimise attrition during the trial [24]. This should also ensure that participants are providing informed consent and are fully committed and motivated to take part in the study before randomisation.

Participants who express an interest in the study, either through self-referral or team referral routes, will be contacted by the IBPI team by telephone to complete a pre-screen form to assess their likely eligibility and be given information regarding the study. If they confirm their wish to participate they will be emailed a participant information pack and web link directing them to consent online. The online link will take the participant to the study website consent screens, each presenting a single clause of the consent form. A consent button is used for each page to ensure there is clear consent to the study as a whole before proceeding to a final screen in which the individual provides their name, email address and telephone number to permit data validation, confirm identity and facilitate contact as required to support assessments. Online registration data will be stored on the secure study database. Once registered, each participant will be provided with login information for both bipolar and parenting modules.

### Inclusion/exclusion criteria

Potential participants must meet the inclusion criteria of:

- Structured Clinical Interview for DSM-IV Axis I Disorders (SCID) verified diagnosis of bipolar disorder (I, II or Not Otherwise Specified) [27]
- Have at least one child aged 3 to 10 years
- Have an average of at least 10 h face-to-face contact with this child each week
- Have access to the internet
- Sufficient understanding of written and spoken English in order to provide consent, engage with interviews, complete measures and use the intervention

Exclusion criteria:

- Absence of inclusion criteria
- Have a primary diagnosis of alcohol or other substance misuse
- Unable or unwilling to provide informed consent
- Parent currently receiving an evidence based structured parenting intervention and/or intensive psychotherapy
- The index child currently in receipt of intensive structured psychological therapy and/or currently the subject of child protection proceedings

### Outcome measures

To evaluate the feasibility and acceptability of delivering the IBPI intervention the following data will be evaluated: levels of recruitment into the trial; retention of participants in both arms of the study and adherence to and completion of the intervention (website useage).

Structured Clinical Interview for DSM-IV at baseline will be used to confirm bipolar diagnosis and to provide sociodemographic information. Measures of clinical outcome will be recorded at baseline and follow-ups to provide an indication of the effectiveness of the intervention on child behaviour, and parenting skills and confidence. Secondary outcomes are levels of self-reported affective mood symptoms and time to mood episodes and family functioning. All observer-rated follow-up assessments are conducted by telephone, all self-report assessments are completed online.

### Feasibility and acceptability outcomes

The primary outcome of the study is the feasibility and acceptability of IBPI. Feasibility will be measured by recruitment to target and retention to follow-up as well as absence of untoward incidents associated with IBPI. Acceptability will be measured by adherence to intervention through website usage data (pattern and frequency of module access).

### Measurement of primary clinical outcomes

Hypotheses are that IBPI will improve i) Child Behaviour as perceived by the parents and measured by the Strengths and Difficulties Questionnaire (SDQ) [28] and Eyberg Child Behavior Inventory (ECBI) [29]; and ii) Parenting Skills and confidence as measured by the Parenting Scale (PS3) [30], Parenting Sense of Competency Scale (PSOC) [31,32] and Parenting Stress Index (PSI) [33].

### Measurement of secondary outcomes

Hypotheses are that IBPI will improve; i) Self-reported symptoms of mania and depression (Internal States Scale (ISS) [34] Centre for Epidemiologic Studies Depression Scale (CES-D) [35] and the Altman Rating Scale (AMRS) [36]; ii) Observer rated time to relapse and mood symptoms (Structured Clinical Interview for DSM-IV-TR AXIS 1 DISORDERS - LIFE (SCID-LIFE) [37], Hamilton Depression Rating Scale (HAM-D) [38], Bech-Rafaelsen Mania Scale (MAS) [39]; iii) family coherence measured by the Confusion, Hubbub and Order Scale (CHAOS) [40].

### Schedule of assessments

The first follow up assessment occurs 16 weeks from randomisation. See Table 1 for more details on specific assessments and follow up periods. Online assessment measures will be delivered via the LimeSurvey open source questionnaire system (https://www.limesurvey.org/en/). At each online assessment point, participants will be sent an email asking them to follow the link provided to complete the measures. The research team web developer (RP) will monitor the completion of questionnaires and send reminder emails on a regular basis. In the event that email contacts do not elicit a response, the team will make telephone calls to facilitate the completion of measures where such support may be required.

**Table 1:** Schedule of assessments

Time point	Type of outcome	Outcome measures	Measuring change in
• Baseline	Feasibility	Recruitment and retention to follow-up	N/A
• 16, 24, 36, 48-weeks post randomization			N/A
• During intervention		Adherence to intervention and website usage	
• 16-weeks post randomization	Acceptability		N/A
• Baseline,	Primary Clinical	SDQ	Child Behaviour
• End of intervention (16 weeks), and		ECBI	Child Behaviour
		PS3	Parenting
• 24-, 36-, and 48- weeks post randomization.		PSOC	Parenting
		PSI	Parenting
Via online survey	Secondary Clinical	ISS	Parent symptoms
		CES-D	Parent symptoms
		AMRS	Parent symptoms
		CHAOS	Parent symptoms
• Baseline,	Secondary Clinical	SCID-LIFE	Parent symptoms
• 24- and 48-weeks post randomization.		HAM-D	Parent symptoms
		MAS	Parent symptoms
Via phone interview with RA			

### Integrated bipolar parenting intervention

An 8 module intervention will be provided to parents offering self-management strategies for bipolar disorder (see Table 2). This is programmed based on the Drupal open source content management system written in PHP with a MySQL database. All modules were developed in partnership with service users with lived experience of bipolar disorder and parenting. The modules listed below cover a range of aspects of living with bipolar disorder all providing a mixture of information, video clips from professionals and service users and self-evaluation exercises. Each module topic is presented in the context of both bipolar disorder and parenting – for instance the ‘benefits and challenges’ module looks at how bipolar mood experiences can have both positive and negative impacts on parenting.

**Table 2:** List of IBPI modules

	Bipolar Modules	Triple P Modules
1	What is bipolar disorder?	What is positive parenting?
2	Benefits and challenges	Encouraging desirable behaviour
3	Managing emotions	Teaching new skills and behaviour
4	Knowing yourself	Managing misbehaviour
5	Mood monitoring	Dealing with disobedience
6	Playing to your strengths	Preventing problems by planning ahead
7	Planning for yourself	Making shopping fun
8	Finding support and final thoughts	Raising confident competent kids

After each bipolar module the participant is offered access to the linked parenting module from the Level 4 Triple P online intervention [24]. Both elements of the IBPI intervention share interactive and multimedia features including: video clips, interactive exercises, self-evaluation exercises. Both also share a normalising, self‐regulatory focus to specifically avoid stigmatising or blaming participants.

Each module will typically last around 30 mins and it is anticipated that parents will cover around 1 module per week. Each parent allocated to the intervention arm of the trial has access to the intervention for 16 weeks. Participants are offered modules in the order noted above as they follow logically from one to the next. However, this order of module completion is not obligatory so participants can select modules as they wish and return to those which they find most useful.

### Analysis

#### Feasibility outcomes

The primary purpose of the study is to evaluate the feasibility and acceptability of delivering IBPI therefore a formal power calculation comparing treatment groups is not required. We anticipate that approximately 60 % of subjects will complete the assessment. With 100 subjects recruited across both treatment arms, this proportion will be estimated with 95 % confidence limts +/−9.6 %. The corresponding limits for the estimated proportion within each treatment arm will be +/− 13.6 %

Feasibility is a binary outcome. A simple logistic regression analysis will be used to estimate the probabilities of completion of follow-up in each group at each assessment point, and the differences between the two. Acceptability will be evaluated by analysis of web useage data. Pattern and frequency of module access will be explored through time to access the site from account creation, number of views of each module, order of viewing of modules and number of specific page views.

#### Clinical and functional outcomes

Clinical and functional outcomes consist of repeated measurements. These will be taken at weeks 0, 16, 24, 36 and 48 following randomisation for most measures, except SCID-LIFE, HAM-D and MAS which will be recorded a 0, 24,and 48 weeks (but assessment of episodes covers all the weeks from the previous assessment). Records on participants who drop out of the study before week 48 will be incomplete. Analysis of measurements and dropout times will include checks that groups are balanced with respect to age, gender, level of education and use of services, and control for these as required, followed by linear modelling with correlated errors for the repeated measurements and linked logistic regression models for the probability of dropout conditional on the repeated measurements prior to dropout [41]. In order to inform the design of a definitive trial, these analyses will investigate which patient characteristics affect the repeated measurement outcomes and/or the probability of dropout.

To determine how well IBPI worked for those who did not drop-out, the main clinical outcome measure is D, the difference in SDQ and PS pre- and post-intervention. The corresponding primary analysis will be a two-sample t-test comparing values of D in the two groups. Post-intervention, it is hypothesised that participants who received IBPI will have a greater D than those receiving assessments only. We will also explore website usage data.

## Discussion

This study will provide important data for the development of a future definitive IBPI trial should the results of this trial look promising. The online parenting intervention IBPI has been developed with experts in positive parenting interventions, experts in the self-management of bipolar disorder and individuals who have experience of bipolar disorder consistent with Mental Health Research Network good practice guidelines [42]. In addition, service user involvement has been a core aspect of the research process, from inception of ideas through to the development of the methodology, outcome measures and the intervention. The intervention itself promotes positive parenting and builds upon skills which parents already possess. There is currently no other known intervention which specifically provides targeted support for parents with bipolar disorder.

Strengths of the study include a focus on an intervention which integrates strategies to aid living well with bipolar disorder in the context of being a parent of young children. It also offers the potential to increase access to evidence-based support for a group who currently have limited access to psychological interventions in general and particularly to interventions which also incorporate consideration of the challenges of parenting. The diverse nature of the research and intervention development team have ensured that the intervention is grounded in lived experience of being a parent with bipolar disorder, with the addition of opinions from experts in the field. The nature of IBPI means that each parent can personalise and tailor their own intervention, allowing them to get the most out of it for their circumstances. The intervention is intended to be both flexible and appealing, acknowledging the issue of engagement. All modules can be started, paused and recommenced when convenient or when feeling able to focus on the content.

The online RCT aims to be as rigorous as a traditional RCT but reducing the impact of potentially confounding variables such as extensive contact with the research team. In line with the definition of Mathieu *et al*. [43] the current trial is *primarily* rather than *fully* on line (as not all of the assessments are completed online). Participation in the trial is from home and observer-rated assessments are made via the telephone consistent with this minimal contact (whilst all self-report measures are completed online). A diagnosis of bipolar disorder is verified at baseline; followed by a series of outcome measures at baseline and then at follow up time points. Evaluating IBPI online makes it possible to recruit participants nationally, and capture outcome data instantaneously. This study is recruiting from across NHS primary and secondary care settings throughout the United Kingdom and through self-referral with a focus on online recruitment. Therefore, the findings should be more representative of a group of parents with bipolar disorder who use the internet for parenting support. This means higher levels of reliability, and validity without the cost implications and resources necessary in traditional RCTs.

The weaknesses of this study would need to be addressed in a later definitive trial. However, parenting in bipolar disorder is an area where little is known about the effects of a targeted intervention. The study allows individuals to be followed up for a period of 48 weeks following the use of the IBPI intervention. Further follow-ups would be helpful to indicate more definitively whether this intervention has a sustained impact on longer term parenting and mood outcomes. As the current focus of this trial is on parents of children from 3 to 10 it will not indicate whether this integrated approach might have potential as children enter teenage years and beyond (when risk for increased psychopathology in offspring is detected [44]).

The online nature of this intervention study means that there is a lack of human contact including the absence of a therapist. This potentially puts more demands on the individual to apply self-management strategies on their own initiative. However, this might also be an advantage for some participants, particularly in the context of evidence that individuals with bipolar disorder typically place high value on autonomy [45]. It has been suggested that computer mediated studies may provide an environment which encourages socially desirable or unreliable responding [46]. However, there is contrasting evidence to suggest that such studies are equally as reliable as traditional methods of outcome data collection [47] and it has been argued that the anonymous online environment allows participants to express their opinions and feelings more freely, thus, reducing unreliable responding when completing outcome measures online [48].

In summary, if the current study indicates that IBPI is an acceptable and feasible intervention, in addition to potential clinical benefits it will be an important step towards developing evidence-based interventions for parents with bipolar disorder that have been lacking until now.
